# Using measures of single‐cell physiology and physiological state to understand organismic aging

**DOI:** 10.1111/acel.12424

**Published:** 2015-11-29

**Authors:** Alexander Mendenhall, Monica Driscoll, Roger Brent

**Affiliations:** ^1^ Division of Basic SciencesFred Hutchinson Cancer Research CenterSeattleWAUSA; ^2^ Department of Molecular Biology and BiochemistryRutgersThe State University of New JerseyPiscatawayNJUSA

**Keywords:** aging, chance, physiology, quantitative microscopy, reporter genes, single‐cell, stochastic

## Abstract

Genetically identical organisms in homogeneous environments have different lifespans and healthspans. These differences are often attributed to stochastic events, such as mutations and ‘epimutations’, changes in DNA methylation and chromatin that change gene function and expression. But work in the last 10 years has revealed differences in lifespan‐ and health‐related phenotypes that are not caused by lasting changes in DNA or identified by modifications to DNA or chromatin. This work has demonstrated persistent differences in single‐cell and whole‐organism physiological states operationally defined by values of reporter gene signals in living cells. While some single‐cell states, for example, responses to oxygen deprivation, were defined previously, others, such as a generally heightened ability to make proteins, were, revealed by direct experiment only recently, and are not well understood. Here, we review technical progress that promises to greatly increase the number of these measurable single‐cell physiological variables and measureable states. We discuss concepts that facilitate use of single‐cell measurements to provide insight into physiological states and state transitions. We assert that researchers will use this information to relate cell level physiological readouts to whole‐organism outcomes, to stratify aging populations into groups based on different physiologies, to define biomarkers predictive of outcomes, and to shed light on the molecular processes that bring about different individual physiologies. For these reasons, quantitative study of single‐cell physiological variables and state transitions should provide a valuable complement to genetic and molecular explanations of how organisms age.

## Background: stochastic events and biological outcomes

Lifespan and its newer (and obscurely begotten; Rivlin, 1992) cousin, healthspan, measurable in units of time (or even Quality Adjusted Life Years; Kaplan, 1993) are prime examples of complex quantitative phenotypes not simply determined by genotype and environment. Genetically identical *Saccharomyces cerevisiae* and *Caenorhabditis elegans* raised in homogeneous environments have different lifespans (Mortimer & Johnston, 1959; Klass, 1977; Kirkwood & Finch, 2002); in both organisms, the difference in the age between the first and last deaths in small populations can be more than fivefold. Similarly, human monozygotic twins, raised in similar environments, have different lifespans (Herskind *et al*., 1996). Genetically identical organisms also show different healthspans. For example, human twins of the same age show differences in various tests of strength (Arden & Spector, 1997; Silventoinen *et al*., 2008). In middle adulthood, some isogenic worms in homogenous environments are immobile, whereas others move around youthfully (Hosono *et al*., 1980; Herndon *et al*., 2002). Moreover, in isogenic populations, interindividual differences in other quantitative phenotypes that measure healthspan increase with age (Kirkwood & Finch, 2000; Herndon *et al*., 2002).

Broadly speaking, the observed variation in lifespan and increased variation in health over time is consistent with the idea that decline in health after maturity and eventual death by old age are the consequence of random events (Kirkwood & Finch, 2000; Hayflick, 2007). In this view, cell and organismic death the end result of the progressive loss of function caused by chains of these events, and the fact that these the multiple events occur randomly causes cells and animals to become more physiologically distinct with age (Martin, 2009). In this review, we emphasize this view in contrast to the idea that events during decline and aging are the result of the workings of an explicit genetic program such that which governs metazoan development.

For lifespan, healthspan, and other complex quantitative phenotypes, researchers normally deploy the word ‘stochastic’ to denote nonenvironmental, nongenetic causes of differences (Kliebenstein, 2011) that they do not otherwise understand. When researchers in the aging field have studied stochastic differences in lifespan and healthspan, they have frequently examined lasting changes caused by mutations (Strehler, 1986; Melov *et al*., 1995, 1999; Dolle & Vijg, 2002; Dolle *et al*., 2002; Vijg & Dolle, 2002; Vijg & Suh, 2013) and ‘epimutations’: (e.g., changes in DNA methylation, Fraga *et al*., 2005; Greer *et al*., 2015; and/or histone modifications, Greer *et al*., 2010). It seems possible that some of this emphasis on mutations and epimutations as causal may have been due to the very power of the methods, some general, some of which were specifically and cleverly devised, allowing detection of aging‐related changes in DNA and chromatin. We imagine that additional kinds of stochastic events that might contribute to aging. For example, consider differential segregation of regulatory molecules or molecular complexes present in small number (Delbrück, 1940, 1945; Spudich & Koshland, 1976) and spontaneous or signal‐triggered changes in conformation of proteins that form aggregates affecting cell function (Cox, 1971; Jarosz *et al*., 2014a,b) or causing cell toxicity (Morley *et al*., 2002; Brignull *et al*., 2006). In that spirit, we review recent technical and conceptual progress that should enable a complementary experimental approach. It has now become possible to observe and quantify changes in physiology and in physiological state in living single cells in intact animals over time. This newfound ability will allow ordering of events and changes of cell state as animals age, definition of the cell‐ and organism‐level consequences of the changes in cell function, and suggest testable hypotheses for the molecular mechanisms that bring these about, without prior assumptions as to causation.

## The complementary concept of physiological states

In this review, we will build on an older, premolecular picture of biological function, one we believe may be particularly relevant to an understanding of aging. In this view, biological systems, including cells and organisms, exist in discrete physiological states. These states are defined by different combinations of observable characteristics that persist over time. Causal events, which include all of the different stochastic events mentioned above, but whose nature does not need to be specified, can induce the system to make a transition from one physiological state to a different state. This new state again tends to persist over time, until transition to another state. The temporal progression of biological systems, during postembryonic‐developmental aging, or in response to defined events that affect function, can thus be viewed as a set of transitions from state to state. In medicine, this view is partly explicit in work of Cannon (Cannon 1929).

Here, we develop and use a framework and vocabulary (Box 1) for describing these physiological states and state transitions. This roots of the framework are work by 19th and early 20th century physicists, who realized that changes in the values of variables representing different properties of dynamical systems over time could define trajectories through a high‐dimensional space (Gibbs, 1902; Nolte, 2010). Subsequently, the use of values of variables representing properties of systems, and the metaphor that these systems described trajectories through high‐dimensional phase spaces or state spaces over time, came to be used in disciplines as diverse as engineering and meteorology (Lorenz, 1963). Significantly, this framework represents and first focuses on of the states, state transitions, and trajectories, and the consequences of these things, rather than on their causes.

Box 1 **Terms and concepts pertinent to the study of physiological states**

- *Variables* describe *phenomena* whose values vary and whose values we can measure.
- *Cell physiological variables* are variables whose values reflect aspects of cell function.
- Values of cell physiological variables can be *continuous*, or *discrete*.
- Cell physiological variables can quantify *states*,* events,* and *processes*.
- Physiological variables that quantify *events* are discrete. For example, the value of a variable called *double‐stranded DNA breaks* would be an integer quantity, and value of a variable called *nuclear membrane integrity* might be 0 or 1.
- Physiological variables that quantify *processes* are typically continuous, or best viewed as continuous, even if integer. For example, the number of protons exported from the cell per unit time is a continuous variable.
- The possible range of values that a physiological variable can assume defines a dimension in a *physiological state space*. A physiological state space has as many dimensions as it has physiological variables.
- Physiological states can be defined arbitrarily, by the researcher, as ranges of values of physiological variables. For example, we might define *extreme acidity* as state defined by a pH between 0 and 6.5.
- Physiological states can also be defined by direct experiment, as *clusters of values of different variables* within a population or over time, that are *close in distance within the physiological state space*.
- When present, such clusters define *basins of attraction* within the state space.
- For individual cells and organisms, changes in the values of physiological variables over time describe *trajectories* in the physiological state space.
- In some cases, over time, the trajectories of cells and organisms described can be considered as movement from one basin of attraction to another.
- Some physiological variables measure, and some physiological states reflect, *cellular pathologies*.
- We refer to a trajectory through physiological state space that traverses multiple states associated with cellular pathologies as a *decline path*. A decline path that ends in cell death is a *failure path*. A decline path that ends in organism death is a *fatality path*.

**Figure 1:**
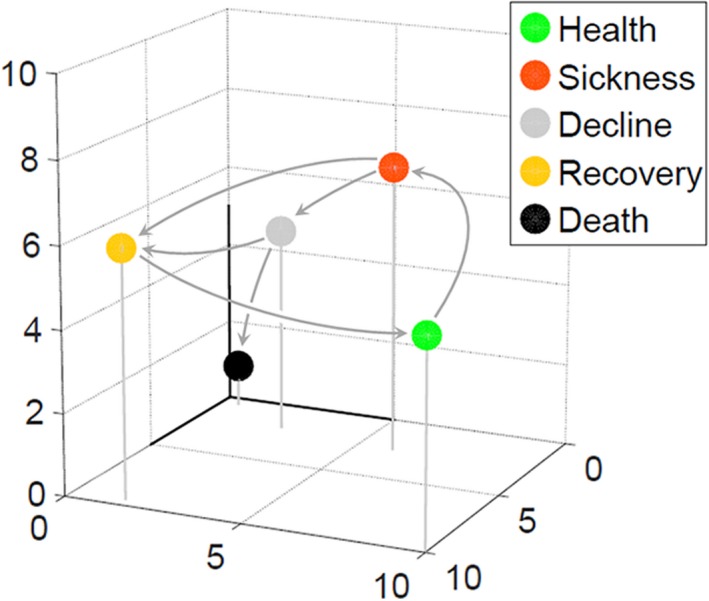
States and trajectories in physiological state space. Hypothetical plot shows values of three physiological variables measured in different individuals at different times. Measurements define clusters of points close in Euclidean distance in this space, here denoted by colored circles. Identification of such clusters is a quantitative means allowing researchers to define, operationally, qualitatively distinct physiological states. In the example here, the different colors correspond to states qualitatively used to describe health and sickness, and arrows indicate transitions between different states observed in different individuals. Values of variables from a given individual over time define a trajectory in this space.

One application of such thinking to whole‐organism physiology came from work in the 1970s by a group of physicians Siegel *et al*. (1974, 1979). They used measurable quantities (e.g., cardiac output, heart rate) in critically ill patients after surgery, and plotted their values in high‐dimensional space. They used ranges of values of these variables to define five prototype states (Siegel *et al*., 1979). For different patients, the values of these variables over time described different, stereotyped trajectories from cluster to cluster, or state to state, as those patients recovered, or as they became sicker and died. Later, Rixen *et al*. (1996) showed that patients in septic shock moved between seven physiological states defined by 17 measured variables, and that different patients followed particular ‘routes’ between these states. Figure 1 shows a general illustration of this concept applied to human disease states. It shows different prototypical states, in a physiological state space defined by only three variables, and potential trajectories described by different patients making transitions between these states. This framework thus naturally represents changes in system function over time.

## Newfound abilities to monitor physiological states in single cells

The trigger for this review is the fact that, enabled by the development of fluorescent proteins, it is now possible to observe and quantify physiological variables in single cells in living organisms over time (Mendenhall *et al*., 2015). Table 1 shows different classes of physiological variables that are now quantifiable by such means. These include variables whose values are continuous and that define ‘classical’ physiological quantities; consider, for example, pH or redox. They also include variables whose values are integer quantities; for example, consider the number of unrepaired double‐stranded DNA ends a cell has (quantified by punctate signal from a fluorescent derivative of the bacteriophage Mu gamma protein; Shee *et al*., 2013). These variables also include measures of quantities only defined by new discovery. Consider, for example, the conceivable single‐cell variables measuring cell nutritional and growth factor status, now definable by live cell measurement of activity of the mTORC1 complex (Table 1). Twenty five years ago, Tor (Brown *et al*., 1994) and the mTORC complexes were unknown, and researchers could not have thought to measure variables that depended on cellular mTORC1 function until the relevant molecules nor their functions has been described. Finally, this also includes ‘systems level’ variables (e.g., **G**, which measures the general ability of a cell to express proteins; Colman‐Lerner *et al*., 2005), which were previously unknown (Table 1). Such variables are computed from measurements of multiple reporters in single cells. Some of the physiological states defined by ranges of values of these variables are consequential (see below).

**Table 1:** Some types of physiological variables now quantifiable in single cells

Type of variable	Example variable	Example quantification methods	Example state terms
Continuous variables defining classical states	pH; glutathione redox	Signal from fluorescent protein derivatives engineered to change emission intensity or emission spectrum on change in measured condition	‘Acidic’; ‘highly reducing’
Discrete or integer variables	Double‐stranded DNA breaks; number of specific mRNAs	Signal from fluorescent protein derivative engineered to form patches on double‐stranded DNA ends (DSEs); signal from fluorescent protein derivative that binds sites in 3′ region of mRNAs transcribed from engineered genes	‘Possessing double‐stranded breaks’; ‘numerous copies of a particular mRNA’
Continuous variables defining other nonclassical states	‘Growth‐factor‐dependent‐mTORC1 status’	Signal from fluorescent protein derivative that changes FRET signal on phosphorylation by mTORC1	‘Highly growth factor stimulated’, ‘rapamycin inhibited’
Continuous variables defining ‘systems level’ states	**G** (gene expression capacity)	Cell's position on a correlated variation axis made by plotting the outputs of two different promoters expressing two different fluorescent proteins	‘High gene expression capacity’

The technical ability to quantify classical and new physiological variables over time is well matched with representation and analysis via a modest generalization and extension of the state space metaphor. We detail this generalization in the Box 1. Five aspects of this generalization merit mention here. First, any given physiological variable may or may not be pertinent to the understanding of a given process, but many quantities are easy to measure, and, once measured, their values defines a coordinates on one axis in a higher dimensional space. Second, a researcher can, at will, define a range of values for a single variable or set of variables as a *physiological state* (e.g., the range of values of pH lower than 6.5 can define a cellular state of *extreme acidity*). Third, even without the guidance of researchers, measurements of physiological variables can themselves define different physiological states. Such states become apparent as clusters of values of variables within given ranges, common to many different cells or organisms, and/or maintained by the same cells or organisms over time. We can refer to either sort of cluster as a *basin of attraction* in the state space. Fourth, changes in the values of these variables over time define trajectories in the state space, including (pertinent to morbidity and aging) *decline paths* and *failure paths* (Box 1). Fifth, the state space representation says nothing about causes (stochastic or otherwise) of the state transitions, nor about mechanisms (molecular or otherwise) by which the values of variables that define given states are maintained. *In this representation, it is the states, state transitions, and trajectories among the states that are the objects of first‐order study – not the particular molecular mechanisms that bring these about*.

We review below a number of ways in which this newly possible study of cell physiological states, state transitions, and trajectories, and their relation to whole‐organism measures of physiology, has the potential to complement existing genetic and molecular pictures of organismic aging, and to guide new research.

## Quantification of reporter outputs in living single cells can define ‘systems level’ physiological states

**Figure 2:**
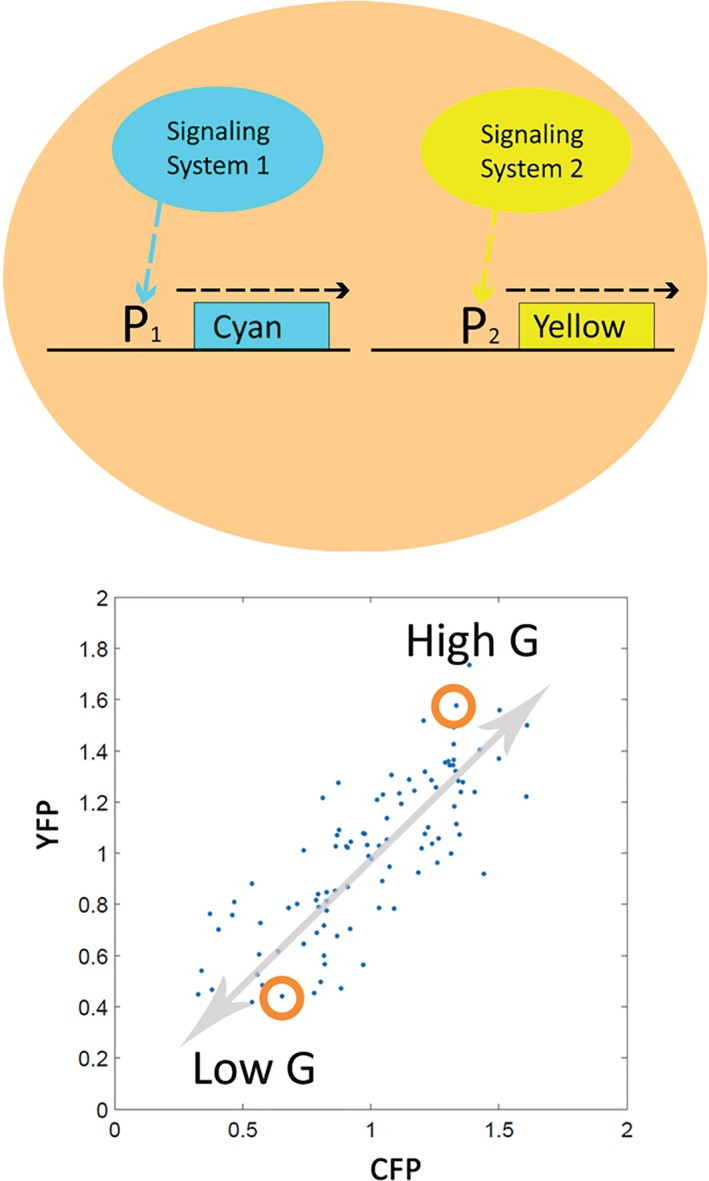
Quantification of a single‐cell physiological variable, **G**, in *Saccharomyces cerevisiae*. Top Panel. A cell containing two reporter genes after (Colman‐Lerner *et al*., 2005). Signaling System 1 activates Promoter 1, P1, which directs synthesis of a cyan fluorescent protein. An unrelated system, Signaling System 2, activates an unrelated promoter, Promoter 2, which directs the synthesis of yellow fluorescent protein. Bottom Panel. Correlated output of the reporter genes in a population of isogenic single cells (Colman‐Lerner *et al*., 2005). Each dot shows YFP and CFP signal from a single cell, quantified by careful light microscopy (Gordon *et al*., 2007; Bush *et al*., 2012). Gray arrow shows correlation line. Correlated variation defines a new single‐cell physiological variable, **G**, a measure of the general ability of each cell to express genes into proteins. Cells with higher correlated expression have higher **G**. Red circles show two cells, one with low **G**, one with high **G**. Cell states defined by this variable persist over many hours. Consequences of a high **G** state include heightened expression of all measured genes, and a more rapid increase in cell volume (Colman‐Lerner *et al*., 2005).

Knowledge of some of the new single‐cell and whole‐organism physiological states did not come from testing of previous hypothesis, but rather directly from consideration of experimental data. One set of insights came from our studies of a particular signal transmission pathway in single yeast cells (Colman‐Lerner *et al*., 2005), the *S. cerevisiae* pheromone response system. These experiments depended on the development of accurate means to quantify reporter signal from cells with low‐measurement error (Gordon *et al*., 2007), including accounting for different rates of fluorophore maturation and inhibition of cell cycle progression to eliminate that source of variability. By measuring fluorescent signal from pheromone‐inducible reporter genes and from control reporter genes, in homogenous cultures of isogenic cells, in which cell cycle progression had been arrested, we quantified different sources of variation in cell signaling and in response. Importantly, only a small proportion of total cell‐to‐cell variation was caused by random fluctuations in gene transcription and translation (‘expression noise’ or ‘intrinsic noise’, **γ**). Instead, cell‐to‐cell variation in signaling and response was dominated by differences in the capacity of individual cells to transmit signals through the pathway (‘pathway capacity’, **P**) and to express proteins from genes (‘expression capacity’, **G**). We encountered **G** when we found that the amount of fluorescent signal from the pheromone‐induced reporter was highly correlated with that from control, constitutive promoters. Cell‐to‐cell variation in the correlated ability of isogenic cells to express different reporters defined cell‐to‐cell differences in **G** (Fig. 2). Cells with high **G** expressed proteins at a higher rate and increased in volume more rapidly. Differences in **G** persisted for many hours. **G** thus defined a hitherto undefined single‐cell physiological variable, and to define cells within a given range of values of **G** as being in a particular, persistent physiological state.

**Figure 3:**
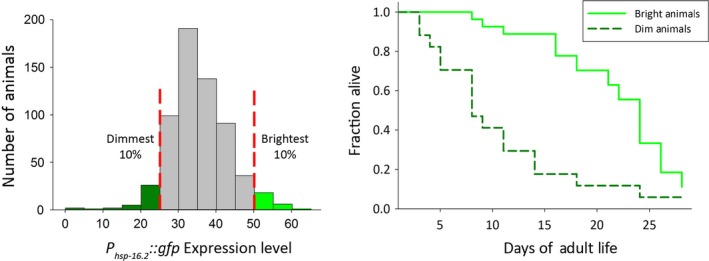
Interindividual differences in gene expression in isogenic populations of C. elegans and differences in lifespan predicted by differences in gene expression. Data replotted from (Mendenhall *et al*., 2012). Left Panel. Distribution of values of reporter expression among animals in the population. Vertical bars show brightest and dimmest 10% of animals. Right Panel. Animals in the highest range of values for gene expression (solid bright green line) live longer than those in the lowest range (dashed darker green line). Therefore, in this example, both high and low ranges of values of the physiological variable, *P*_*hsp‐16.2*_ *‐GFP‐signal*, operationally define distinct and consequential whole‐organism physiological states.

Additional insights came from our work in *C. elegans* (Rea *et al*., 2005; Mendenhall *et al*., 2012; Fig. 3). In 2005, Rea *et al*. studied isogenic worms raised in the same environment that carried a reporter gene in which the *hsp‐16.2* promoter drove synthesis of GFP (here, *P*_*hsp‐16.2*_ *::gfp*). Young adult animals differed greatly in the amount of green fluorescent signal they showed after a heat shock. This difference was consequential: bright green worms lived longer and were more resistant to subsequent stress. The authors suggested that differences in the amount of HSP‐16.2 protein itself were ‘probably not responsible for the observed differences in survival but that the differences in fluorescence probably reflected a hidden, heterogeneous, but now quantifiable, physiological state that dictates the ability of an organism to deal with the rigors of living.’

**Figure 4:**
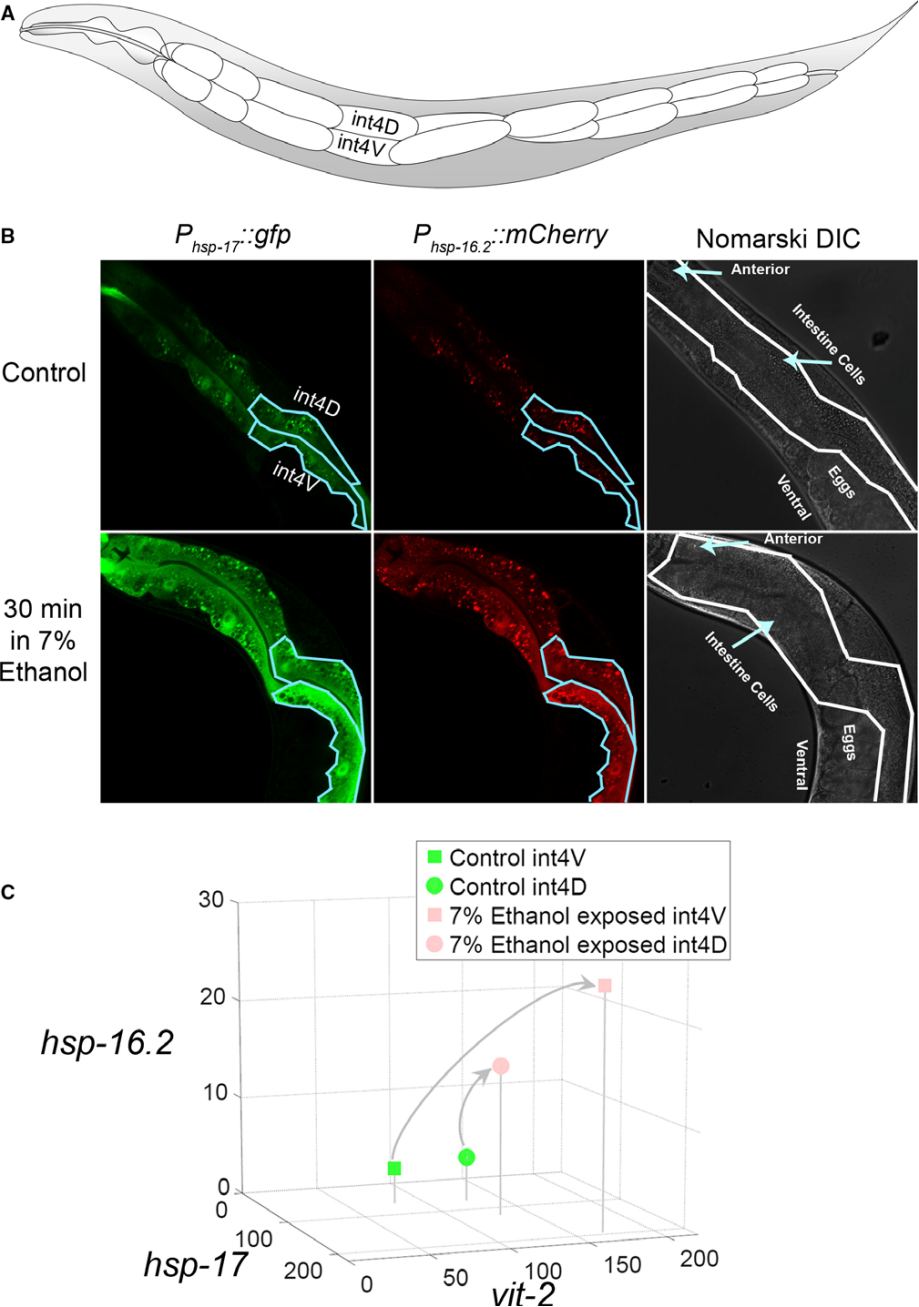
Single‐cell trajectories through physiological state space defined by changes in the values of reporter gene expression. (A) Layout of intestine cells in the adult worm, showing cells int4V and int4D. (B) Induced expression of *P*_*hsp‐17*_ *::gfp* and *P*_*hsp‐16.2*_ *::mCherry* reporters in cells int4V and int4D in control animals and animals treated with an acute stimulus (ethanol exposure). (C) Trajectories of measured int4V and int4D cells from control‐ and ethanol‐exposed animals 16 h after 30 min 7% ethanol exposure, plotted in a three‐dimensional state space whose axes correspond to expression of the *P*_*hsp‐17*_ and *P*_*hsp‐16.2*_ reporters and *P*_*vit‐2*_ reporters.

Our subsequent studies showed that the bulk of the fluorescent signal came from the 20 individual cells (Seewald *et al*., 2010). Subsequent work to develop low measurement error methods for single cells showed that the reporter signal could be quantified in individual cells in anesthetized live animals (Mendenhall *et al*., 2015; Fig. 4). This work confirmed the correlation between the state of high GFP expression in young adulthood and lifespan (Mendenhall *et al*., 2012) and identified a difference in healthspan (Cypser *et al*., 2013); animals in the state defined by high GFP expression in young adulthood showed more motility at a given age. This work thus defined a physiological variable, *P*_*hsp‐16.2*_ *‐GFP‐signal*, whose value in turn defined a physiological state, measurable in single cells, which correlates with healthspan. This state of high expression was heritable (Cypser *et al*., 2013) and may be due to the same organism‐to‐organism differences in **G** observed in *S. cerevisiae*.

## Quantification of existing fluorescent reporter outputs allows single‐cell measurement of numerous additional physiological states

In addition to these ‘systems level’ single‐cell physiological variables, different kinds of reporters now allow quantification of numerous additional physiological variables in living single cells. Important classes of reporters now include:

*Reporters that quantify induction of gene expression by fluorescent protein signal*. In the experiments that correlated the expression of the *P*_*hsp‐16.2*_ *::gfp* reporter with lifespan, quantification of the physiological variable depended on a reporter in which production of a particular inducible promoter drove synthesis of the fluorescent protein. A large number of such ‘promoter fusion’ or ‘transcriptional’ reporters for physiological variables thought to be relevant to aging already exist; for example, reporters for reactive oxygen species in *C. elegans*, in which the *sod‐3* superoxide dismutase promoter drives synthesis of GFP (Henderson *et al*., 2006 and now in single copy – A. M. unpublished). Information on particular transcripts induced under different conditions, to identify particular inducible promoters researchers can use to make new reporters, is now widely available.
*Reporters that quantify induction of gene expression by enabling visualization of mRNAs in vivo*. More recently, complementary technology that enables direct quantification of mRNAs in single cells without use of reporter gene products has been developing rapidly in yeast. These methods quantify fluorescent signal from RNA bacteriophage coat protein – fluorescent protein fusions, which bind repeated binding sequences engineered into the noncoding RNA of the gene whose transcript will be counted (Larson *et al*., 2011; Lenstra & Larson, 2015). We imagine that this direct RNA quantification will be applied to measure outputs from inducible promoters in single cells of *C. elegans* and other multicellular model organisms now used in aging research.
*Reporters that quantify cellular events by changes in the subcellular localization of fluorescent protein signal*. Consider a fusion protein comprised of the *C. elegans* FOXO3 homolog, *daf‐16* (Henderson & Johnson, 2001) and a fluorescent protein moiety. Upon stimulation with insulin‐like growth factors or whole‐animal treatments that induce insulin‐like growth factor signaling, FOXO3/DAF‐16 is excluded from the nucleus (Datta *et al*., 1999; Henderson & Johnson, 2001). The ratio of nuclear localized to cytoplasmic FOXO3 fusion proteins thus provides a real‐time measure of the amount of insulin signaling. Such use of protein movement to different subcellular locations to quantify signaling events precisely is now very well established in yeast and mammalian cells (Datta *et al*., 1999; Cai *et al*., 2008; Baltanas *et al*., 2013; Blaustein *et al*., 2013; Bush & Colman‐Lerner, 2013).
*Reporters that quantify ‘classical’ and ‘new’ physiological variables by changes in emission or excitation fluorescence spectra*. The last 10 years has seen a great deal of work developing fluorescent protein derivatives in which the proteins themselves are the reporters. In these proteins, changes in fluorescent resonance energy transfer in dual fluorophore proteins, or changes in the spectrum of light emitted by single fluorophore proteins, can quantify a number of ‘classical’ cell physiological variables. Incorporation of appropriate localization moieties into such proteins allows them to quantify these variables in different subcellular locations and compartments. ‘Simple’ variables that can be quantified by such fluorescent protein reporters include pH (pHlourin and super ecliptic pHlourins (Miesenbock *et al*., 1998), pHRed (Tantama *et al*., 2011)), redox states (e.g., Grx1‐roGFP2 to quantify GSH/GSSG redox; Gutscher *et al*., 2008), level of H_2_0_2_ (HyPer; Lukyanov & Belousov, 2014), levels of calcium (Zhao *et al*., 2011), ATP/ADP ratio (Berg *et al*., 2009), and the ratio of NAD+/NADH (Hung *et al*., 2011).

As mentioned, the development of sensors that respond to the activity of newfound molecules has also opened the way to definition and measurement, in single cells, of aging‐relevant variables that 20 years ago were unknown. For example, the discovery of the metazoan mTORC1 (Kim *et al*., 2002) complex enabled the development of sensor molecules for its activity (Zhou *et al*., 2015). The development of such sensors makes it possible to define and quantify variables related to mTORC1 activity such as: *nutritional‐mTORC1‐status* and *growth‐factor‐dependent‐mTORC1‐status*. The values of these two new variables would reflect, respectively, cellular nutrient supply, and stimulation by extracellular growth factors, and would change in response to inhibition by drugs such as rapamycin.

## Advances in gene technology and image cytometry support better live‐cell measurement of states

At the same time as the above developments demonstrate the relatively broad scope now available to design reporters to quantify additional physiological variables, other technical advances have made it easier to assemble DNA for new reporter constructs, to make transgenic organisms that contain the constructs, and quantify reporter output. Rapid assembly of reporter constructs is aided by improved *in vitro* DNA assembly methods and widespread adoption of construction by homologous recombination in yeast (Sands & Brent, 2015). Rapid construction of transgenic strains with well‐behaved reporters in *C. elegans* has been greatly aided by the development of methods that allow integration of reporters at defined chromosomal sites (Frokjaer‐Jensen *et al*., 2008). Moreover, in all organisms, construction of well‐defined reporter strains has been greatly aided by CRISPR/Cas9 methods that use engineered proteins and RNAs to direct DNA binding, nicking, and cleavage to defined sites throughout the genome (Frokjaer‐Jensen, 2013; Doudna & Charpentier, 2014).

Advances in microscopy and improvement in protein fluorophores have increased the sensitivity and accuracy with which physiological variables can be quantified. Key improvements include increasing use of fluorescent lifetime microscopy to quantify physiological variables (e.g., NADH) (Stringari *et al*., 2011) and to increase signal to noise (Zeug *et al*., 2012), and steady improvement in means to illuminate the volume to be interrogated (Gao *et al*., 2014; Winter & Shroff, 2014; Chen *et al*., 2014). They also include lower technology low‐measurement‐error microscopic methods to facilitate longitudinal measurement of the same set of variables from designated cells in an organism as an organism ages (Mendenhall *et al*., 2015). Meanwhile, steady increases in the numbers of different emission maxima, excitation maxima; and fluorescent lifetimes of fluorescent proteins (Chudakov *et al*., 2010; Sands *et al*., 2014) have increased the numbers of distinguishable signals that can be quantified from the same cell.

## Maps of trajectories in state space might help frame quantitative single‐cell physiological measurements as biological explanations

**Figure 5:**
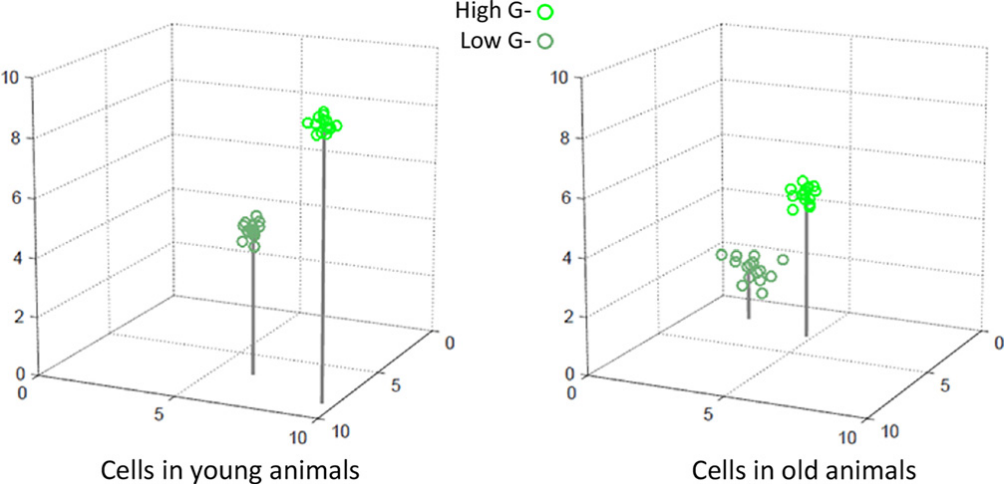
Hypothetical single‐cell trajectories in a three‐dimensional state space during aging. Figure shows values for three physiological variables in a particular cell in young and old *Caenorhabditis elegans*. Young animals and old animals are in two groups collected by sorting as in Fig. 3: long‐lived, bright, high **G** and short‐lived, dim, low **G**. In this example, values for all three variables were lower in older animals. Moreover, cell‐to‐cell variation in the measured variables was higher in older animals, with the highest variation in the dim, low **G** group. In practice, future experiments will likely measure more than three variables per cell and statements about the trajectories defined by changes in values will be based not on inspection of plots but on computed changes in position in the appropriate high‐dimensional space.

The above technological developments will enable collection of dozens of physiological variables from single cells in living animals. They will also allow its interpretation within the conceptual framework of high‐dimensional physiological state space. This framework provides means to view temporal progression, and so becomes more powerful when coupled to longitudinal measurements of the same cell or organism over time. Figure 4 shows a simple example of this, the collection (A and B) and plotting of (C) three variables from two cells in the intestine, before and after exposure of animals to ethanol. Figure 5 shows a more complex, hypothetical example. In this example, the difference in measured physiological variables for a particular cell in different animals increases with age. In this example, the increased variation is most marked in animals in a low‐G physiological state.

For aging research, we believe this picture of the movement of cells in trajectories through physiological state space might be particularly appropriate. There are a number of reasons for our belief. One is that understanding of aging has developed sufficiently to permit listing of a finite number of processes believed to contribute to it (National Institute on Aging 2011; Lopez‐Otin *et al*., 2013). Defining and enumerating (are there few, or many?) common decline and failure paths in different cell types will allow ordering of their steps with respect to these known processes. A second is that longitudinal physiological studies of cells in aging organisms will enable experimentation (both hypothesis‐directed and discovery‐driven) to understand single‐cell consequences of genetic and pharmacological interventions already known to alter whole‐organism lifespan and healthspan. A third is to enable and track experimentation designed to suggest or test hypotheses for additional molecules and molecular events responsible for entry into, maintenance within, and exit from, physiological states. Should additional molecular mechanisms be identified, researchers can use those to devise directed pharmacological or genetic interventions to manipulate the probability of state transitions, for purposes of prevention and therapy.

## Single‐cell physiological measurements should aid understanding of whole‐organism phenotypes

We should be explicit as to how measurements of single‐cell variables might inform studies of whole‐organism physiology. The first way is that live cell measurements are nondestructive, and allow correlation of their values with whole‐organism measurements at some later point. A second is that many of the variables described here, such as redox, or hypoxia, are quantified in single cells because that is the only way, or the most accurate way, to measure them. Values are then averaged from dozens or hundreds of cells to permit assertions about the physiological state of the cell population or whole organ. A third is the ability to learn more than is revealed by population averages. It has long been clear (Ferrell & Machleder, 1998) and is now a truism, that measurements from large numbers of single cells can reveal heterogeneity in values not apparent from whole‐population (or whole organ) measures. Revealed heterogeneity (Bahar *et al*., 2006) may be particularly valuable to aging researchers, whose work (when it does not directly address mortality) is still particularly concerned with phenomena of pathology and morbidity. For example, suppose that the value of a particular variable was zero in some cells (widely distributed or spatially grouped) within an organ, and cells with a zero value were known to be on a trajectory associated with a particular kind of necrotic death. Such knowledge might provide insights into possible causes of organ failure not apparent from existing pathological criteria or from population measurements. Such quantitative knowledge might allow operational definition of state transitions now articulated in qualitative terms like ‘tipping points’ (An *et al*., 2012).

That said, we realize that defining relationships between the cell level variables associated with aging‐related states and the organism‐level variables that measure organismic health and lifespan will not always be easy. For example, in our own work with aging isogenic worms cultured in the same environments, we observed three motility response states (Herndon *et al*., 2002). When touched, animals in state A moved sinusoidally away from a touch stimulus. Animals in state B reacted, but moved away nonsinusoidally, and only when prodded with a wire. Animals in state C did not move away from the stimulus but twitched at heads and/or tails. Animals in states B and C animals showed correlated changes in muscle cell ultrastructure. All animals followed the same trajectory (state A to state B or C, state B to state C and then to death), but the timing of state transitions and time in each state differed among individuals. These experiments thus defined whole‐organism physiological states associated with decline in function, and demonstrated interindividual differences in the timing of state transitions that were not caused by differences in the animal's genetics or known environment and that correlated with changes in muscle cell ultrastructure in fixed cells from killed animals. But there the analysis stopped. No matter the sorts of relationship we might be interested in elucidating – whether temporal, causal, molecular, or simply ordinal, the linkage between the whole‐organism phenotype and the changes in fixed cells still remains unclear.

The obvious forward path is to combine single‐cell and whole‐animal measures in living animals over time. For example, in aging *C. elegans*, an increasing number of intestine cells lose DAPI‐stainable DNA, whereas in *daf‐2* mutants, which live longer, more intestine cells retain DAPI‐stainable DNA (McGee *et al*., 2011). At the moment, no relationship, even a temporal or ordinal one, between intestine cell DNA loss and organismic death has been defined. Clearly, definition of such relationships must precede deeper explanations. Such deeper explanations will then require coupling knowledge of cell biology and with deep understanding of the biology of the whole organism (or, in humans, clinical insight).

Although it will be difficult, we confess to finding the idea that investigators may soon possess numerous single‐cell level physiological variables to relate to organism‐level variables to be exciting. Such knowledge will aid hypothesis formation, and so guide molecular studies of how these states might exert their effects on those particular cell biological processes now viewed as fundamental to aging (National Institute on Aging 2011; Lopez‐Otin *et al*., 2013). As mentioned, such knowledge should therefore help to suggest new molecular targets for pharmacological intervention or germline genetic manipulation. But even before such work could suggest targets, knowledge of the, values of physiological variables may help stratify health and disease states now thought to be the same. Studies in model organisms may define variables that affect the probability of particular state transitions; such variables will define new ‘biomarkers’ (Baker & Sprott, 1988) predictive of future outcomes. In humans, stratification via biomarker assay based on single‐cell physiological variables might eventually guide preventive and therapeutic interventions tailored to the physiological states of particular patients. Put into different words, for individual patients, assay of physiological variables would provide a personalized data type to complement the personalized genomic and other ‘big‐data’ types (Council, 2011) that are now envisioned as the basis for the development of a ‘precision medicine’ (Handelsman, 2015).

## Physiological understanding as a complement to genetic and molecular understanding

We write this 15 years into the 21st century. During the previous century, the study of aging made real progress. A large contribution to this increased understanding came from advances in genetic concepts and methods. These allowed controlled experiments to study the consequences of experimental interventions in populations of genetically identical organisms (Pearl, 1928), and established lifespan and other defined phenomena associated with health as quantitative phenotypes. Later genetics, used in concert with the tools of molecular biology, allowed isolation of genes that affected lifespan (Klass, 1983; Friedman & Johnson, 1988), and analysis of how the gene products caused the phenotypes (Ogg *et al*., 1997). By analogy, advances in 21st century methods that allow rigorous definition of cell physiological states may now aid understanding of additional complex phenotypes pertinent to organismic health and lifespan. The prospects for using concepts and methods from near‐future physiology to understand and eventually manipulate longevity and health now seem bright.

## Conflict of interest

None declared.

## Funding

No funding information provided.

## References

[acel12424-bib-0001] An G , Nieman G , Vodovotz Y (2012) Toward computational identification of multiscale “tipping points” in acute inflammation and multiple organ failure. Ann. Biomed. Eng. 40, 2414–2424. 10.1007/s10439-012-0565-9 22527009

[acel12424-bib-0002] Arden NK , Spector TD (1997) Genetic influences on muscle strength, lean body mass, and bone mineral density: a twin study. J. Bone Miner. Res. 12, 2076–2081. 10.1359/jbmr.1997.12.12.2076 9421240

[acel12424-bib-0003] Bahar R , Hartmann CH , Rodriguez KA , Denny AD , Busuttil RA , Dolle ME , Calder RB , Chisholm GB , Pollock BH , Klein CA , Vijg J (2006) Increased cell‐to‐cell variation in gene expression in ageing mouse heart. Nature 441, 1011–1014. 10.1038/nature04844 16791200

[acel12424-bib-0004] Baker GT III , Sprott RL (1988) Biomarkers of aging. Exp. Gerontol. 23, 223–239. 10.1016/0531-5565(88)90025-3 3058488

[acel12424-bib-0005] Baltanas R , Bush A , Couto A , Durrieu L , Hohmann S , Colman‐Lerner A (2013) Pheromone‐induced morphogenesis improves osmoadaptation capacity by activating the HOG MAPK pathway. Sci. Signal. 6, ra26. 10.1126/scisignal.2003312 PMC3701258 23612707

[acel12424-bib-0006] Berg J , Hung YP , Yellen G (2009) A genetically encoded fluorescent reporter of ATP:ADP ratio. Nat. Methods 6, 161–166. 10.1038/nmeth.1288 PMC2633436 19122669

[acel12424-bib-0007] Blaustein M , Perez‐Munizaga D , Sanchez MA , Urrutia C , Grande A , Risso G , Srebrow A , Alfaro J , Colman‐Lerner A (2013) Modulation of the Akt pathway reveals a novel link with PERK/eIF2alpha, which is relevant during hypoxia. PLoS One 8, e69668. 10.1371/journal.pone.0069668 PMC3726764 23922774

[acel12424-bib-0008] Brignull HR , Moore FE , Tang SJ , Morimoto RI (2006) Polyglutamine proteins at the pathogenic threshold display neuron‐specific aggregation in a pan‐neuronal *Caenorhabditis elegans* model. J. Neurosci. 26, 7597–7606. 10.1523/JNEUROSCI.0990-06.2006 PMC6674286 16855087

[acel12424-bib-0009] Brown EJ , Albers MW , Shin TB , Ichikawa K , Keith CT , Lane WS , Schreiber SL (1994) A mammalian protein targeted by G1‐arresting rapamycin‐receptor complex. Nature 369, 756–758. 10.1038/369756a0 8008069

[acel12424-bib-0010] Bush A , Colman‐Lerner A (2013) Quantitative measurement of protein relocalization in live cells. Biophys. J . 104, 727–736. 10.1016/j.bpj.2012.12.030 PMC3566451 23442923

[acel12424-bib-0011] Bush A , Chernomoretz A , Yu R , Gordon A , Colman‐Lerner A . (2012) Using Cell‐ID 1.4 with R for microscope‐based cytometry. Curr Protoc Mol Biol 100, 14.18.1–14.18.26; Chapter 14: Unit 14 18. 10.1002/0471142727.mb1418s100 PMC3485637 23026908

[acel12424-bib-0012] Cai L , Dalal CK , Elowitz MB (2008) Frequency‐modulated nuclear localization bursts coordinate gene regulation. Nature 455, 485–490. 10.1038/nature07292 PMC2695983 18818649

[acel12424-bib-0013] Cannon WB (1929) Organization for physiological homeostasis. Physiol. Rev. 9, 399–431.

[acel12424-bib-0500] Chen BC , Legant WR , Wang K , Shao L , Milkie DE , Davidson MW , Janetopoulos C , Wu XS , Hammer JA 3rd , Liu Z , English BP , Mimori‐Kiyosue Y , Romero DP , Ritter AT , Lippincott‐Schwartz J , Fritz‐Laylin L , Mullins RD , Mitchell DM , Bembenek JN , Reymann AC , Bohme R , Grill SW , Wang JT , Seydoux G , Tulu US , Kiehart DP , Betzig E (2014) Lattice light‐sheet microscopy: imaging molecules to embryos at high spatiotemporal resolution. Science 346, 1257998. 10.1126/science.1257998 PMC4336192 25342811

[acel12424-bib-0014] Chudakov DM , Matz MV , Lukyanov S , Lukyanov KA (2010) Fluorescent proteins and their applications in imaging living cells and tissues. Physiol. Rev. 90, 1103–1163. 10.1152/physrev.00038.2009 20664080

[acel12424-bib-0015] Colman‐Lerner A , Gordon A , Serra E , Chin T , Resnekov O , Endy D , Pesce CG , Brent R (2005) Regulated cell‐to‐cell variation in a cell‐fate decision system. Nature 437, 699–706. 10.1038/nature03998 16170311

[acel12424-bib-0016] Council NR (2011) Toward precision medicine: building a knowledge network for biomedical research and a new taxonomy of disease [WWW doucment]. URL http://www.nap.edu/catalog/13284/toward-precision-medicine-building-a-knowledge-network-for-biomedical-research [accessed on May 5, 2015]. 22536618

[acel12424-bib-0017] Cox BS (1971) A recessive lethal super‐suppressor mutation in yeast and other psi phenomena. Heredity (Edinb) 26, 211–232. 10.1038/hdy.1971.28 5286385

[acel12424-bib-0018] Cypser JR , Wu D , Park SK , Ishii T , Tedesco PM , Mendenhall AR , Johnson TE (2013) Predicting longevity in *C. elegans*: fertility, mobility and gene expression. Mech. Ageing Dev. 134, 291–297. 10.1016/j.mad.2013.02.003 23416266

[acel12424-bib-0019] Datta SR , Brunet A , Greenberg ME (1999) Cellular survival: a play in three Akts. Genes Dev. 13, 2905–2927. 10.1101/gad.13.22.2905 10579998

[acel12424-bib-0020] Delbrück M (1940) Statistical fluctuations in autocatalytic reactions. J. Chem. Phys. 8, 120–124.

[acel12424-bib-0021] Delbrück M (1945) The burst size distribution in the growth of bacterial viruses (bacteriophages). J. Bacteriol. 50, 131–135. 10.1128/JB.50.2.131-135.1945 20989330

[acel12424-bib-0022] Dolle ME , Vijg J (2002) Genome dynamics in aging mice. Genome Res. 12, 1732–1738. 10.1101/gr.125502 PMC187544 12421760

[acel12424-bib-0023] Dolle ME , Snyder WK , Dunson DB , Vijg J (2002) Mutational fingerprints of aging. Nucleic Acids Res. 30, 545–549. 10.1093/nar/30.2.545 PMC99828 11788717

[acel12424-bib-0024] Doudna JA , Charpentier E (2014) Genome editing. The new frontier of genome engineering with CRISPR‐Cas9. Science 346, 1258096. 10.1126/science.1258096 25430774

[acel12424-bib-0025] Ferrell JE Jr , Machleder EM (1998) The biochemical basis of an all‐or‐none cell fate switch in Xenopus oocytes. Science 280, 895–898. 10.1126/science.280.5365.895 9572732

[acel12424-bib-0026] Fraga MF , Ballestar E , Paz MF , Ropero S , Setien F , Ballestar ML , Heine‐Suner D , Cigudosa JC , Urioste M , Benitez J , Boix‐Chornet M , Sanchez‐Aguilera A , Ling C , Carlsson E , Poulsen P , Vaag A , Stephan Z , Spector TD , Wu YZ , Plass C , Esteller M (2005) Epigenetic differences arise during the lifetime of monozygotic twins. Proc. Natl Acad. Sci. USA 102, 10604–10609. 10.1073/pnas.0500398102 PMC1174919 16009939

[acel12424-bib-0027] Friedman DB , Johnson TE (1988) A mutation in the age‐1 gene in *Caenorhabditis elegans* lengthens life and reduces hermaphrodite fertility. Genetics 118, 75–86. 8608934 10.1093/genetics/118.1.75 PMC1203268

[acel12424-bib-0028] Frokjaer‐Jensen C (2013) Exciting prospects for precise engineering of *Caenorhabditis elegans* Genomes with CRISPR/Cas9. Genetics 195, 635–642. 10.1534/genetics.113.156521 PMC3813854 24190921

[acel12424-bib-0029] Frokjaer‐Jensen C , Davis MW , Hopkins CE , Newman BJ , Thummel JM , Olesen SP , Grunnet M , Jorgensen EM (2008) Single‐copy insertion of transgenes in *Caenorhabditis elegans* . Nat. Genet. 40, 1375–1383. 10.1038/ng.248 PMC2749959 18953339

[acel12424-bib-0030] Gao L , Shao L , Chen BC , Betzig E (2014) 3D live fluorescence imaging of cellular dynamics using Bessel beam plane illumination microscopy. Nat. Protoc. 9, 1083–1101. 10.1038/nprot.2014.087 24722406

[acel12424-bib-0031] Gibbs JW (1902) Elementary Principles in Statistical Mechanics. London: Arnold.

[acel12424-bib-0032] Gordon A , Colman‐Lerner A , Chin TE , Benjamin KR , Yu RC , Brent R (2007) Single‐cell quantification of molecules and rates using open‐source microscope‐based cytometry. Nat. Methods 4, 175–181. 10.1038/nmeth1008 17237792

[acel12424-bib-0033] Greer EL , Maures TJ , Hauswirth AG , Green EM , Leeman DS , Maro GS , Han S , Banko MR , Gozani O , Brunet A (2010) Members of the H3K4 trimethylation complex regulate lifespan in a germline‐dependent manner in *C. elegans* . Nature 466, 383–387. 10.1038/nature09195 PMC3075006 20555324

[acel12424-bib-0034] Greer EL , Blanco MA , Gu L , Sendinc E , Liu J , Aristizabal‐Corrales D , Hsu CH , Aravind L , He C , Shi Y (2015) DNA Methylation on N(6)‐Adenine in *C. elegans* . Cell 161, 868–878. 10.1016/j.cell.2015.04.005 PMC4427530 25936839

[acel12424-bib-0035] Gutscher M , Pauleau AL , Marty L , Brach T , Wabnitz GH , Samstag Y , Meyer AJ , Dick TP (2008) Real‐time imaging of the intracellular glutathione redox potential. Nat. Methods 5, 553–559. 10.1038/nmeth.1212 18469822

[acel12424-bib-0036] Handelsman J (2015) Precision medicine: improving health and treating disease [WWW document]. URL https://www.whitehouse.gov/blog/2015/01/21/precision-medicine-improving-health-and-treating-disease [accessed on May 5, 2015].

[acel12424-bib-0037] Hayflick L (2007) Entropy explains aging, genetic determinism explains longevity, and undefined terminology explains misunderstanding both. PLoS Genet. 3, e220. 10.1371/journal.pgen.0030220 PMC2134939 18085826

[acel12424-bib-0038] Henderson ST , Johnson TE (2001) daf‐16 integrates developmental and environmental inputs to mediate aging in the nematode *Caenorhabditis elegans* . Curr. Biol. 11, 1975–1980. 10.1016/s0960-9822(01)00594-2 11747825

[acel12424-bib-0039] Henderson ST , Bonafe M , Johnson TE (2006) daf‐16 protects the nematode *Caenorhabditis elegans* during food deprivation. J. Gerontol. A Biol. Sci. Med. Sci. 61, 444–460. 10.1093/gerona/61.5.444 16720740

[acel12424-bib-0040] Herndon LA , Schmeissner PJ , Dudaronek JM , Brown PA , Listner KM , Sakano Y , Paupard MC , Hall DH , Driscoll M (2002) Stochastic and genetic factors influence tissue‐specific decline in ageing *C. elegans* . Nature 419, 808–814. 10.1038/nature01135 12397350

[acel12424-bib-0041] Herskind AM , McGue M , Holm NV , Sorensen TI , Harvald B , Vaupel JW (1996) The heritability of human longevity: a population‐based study of 2872 Danish twin pairs born 1870–1900. Hum. Genet. 97, 319–323. 10.1007/BF02185763 8786073

[acel12424-bib-0042] Hosono R , Sato Y , Aizawa SI , Mitsui Y (1980) Age‐dependent changes in mobility and separation of the nematode *Caenorhabditis elegans* . Exp. Gerontol. 15, 285–289. 10.1016/0531-5565(80)90032-7 7409025

[acel12424-bib-0043] Hung YP , Albeck JG , Tantama M , Yellen G (2011) Imaging cytosolic NADH‐NAD(+) redox state with a genetically encoded fluorescent biosensor. Cell Metab. 14, 545–554. 10.1016/j.cmet.2011.08.012 PMC3190165 21982714

[acel12424-bib-0044] Jarosz DF , Brown JC , Walker GA , Datta MS , Ung WL , Lancaster AK , Rotem A , Chang A , Newby GA , Weitz DA , Bisson LF , Lindquist S (2014a) Cross‐kingdom chemical communication drives a heritable, mutually beneficial prion‐based transformation of metabolism. Cell 158, 1083–1093. 10.1016/j.cell.2014.07.025 PMC4424051 25171409

[acel12424-bib-0045] Jarosz DF , Lancaster AK , Brown JC , Lindquist S (2014b) An evolutionarily conserved prion‐like element converts wild fungi from metabolic specialists to generalists. Cell 158, 1072–1082. 10.1016/j.cell.2014.07.024 PMC4424049 25171408

[acel12424-bib-0046] Kaplan RM (1993) Quality of life assessment for cost/utility studies in cancer. Cancer Treat. Rev. 19 Suppl A, 85–96. 10.1016/0305-7372(93)90061-u 7679324

[acel12424-bib-0047] Kim DH , Sarbassov DD , Ali SM , King JE , Latek RR , Erdjument‐Bromage H , Tempst P , Sabatini DM (2002) mTOR interacts with raptor to form a nutrient‐sensitive complex that signals to the cell growth machinery. Cell 110, 163–175. 10.1016/s0092-8674(02)00808-5 12150925

[acel12424-bib-0048] Kirkwood TB , Finch CE (2000) Chance, Development, and Aging. New York: Oxford University Press.

[acel12424-bib-0049] Kirkwood TB , Finch CE (2002) Ageing: the old worm turns more slowly. Nature 419, 794–795. 10.1038/419794a 12397339

[acel12424-bib-0050] Klass MR (1977) Aging in the nematode *Caenorhabditis elegans*: major biological and environmental factors influencing life span. Mech. Ageing Dev. 6, 413–429. 10.1016/0047-6374(77)90043-4 926867

[acel12424-bib-0051] Klass MR (1983) A method for the isolation of longevity mutants in the nematode *Caenorhabditis elegans* and initial results. Mech. Ageing Dev. 22, 279–286. 10.1016/0047-6374(83)90082-9 6632998

[acel12424-bib-0052] Kliebenstein DJ (2011) The quantitative genetics of phenotypic error or uniformity. Front. Genet. 2, 59. 10.3389/fgene.2011.00059 PMC3268612 22303354

[acel12424-bib-0053] Larson DR , Zenklusen D , Wu B , Chao JA , Singer RH (2011) Real‐time observation of transcription initiation and elongation on an endogenous yeast gene. Science 332, 475–478. 10.1126/science.1202142 PMC3152976 21512033

[acel12424-bib-0054] Lenstra TL , Larson DR (2015) Single molecule mRNA detection in live yeast. Curr. Protoc. Mol. Biol., in press. 10.1002/0471142727.mb1424s113 PMC4835805 27110320

[acel12424-bib-0055] Lopez‐Otin C , Blasco MA , Partridge L , Serrano M , Kroemer G (2013) The hallmarks of aging. Cell 153, 1194–1217. 10.1016/j.cell.2013.05.039 PMC3836174 23746838

[acel12424-bib-0056] Lorenz EN (1963) Deterministic nonperiodic flow. J. Atmospheric Sci. 20, 130–141.

[acel12424-bib-0057] Lukyanov KA , Belousov VV (2014) Genetically encoded fluorescent redox sensors. Biochim. Biophys. Acta 1840, 745–756. 10.1016/j.bbagen.2013.05.030 23726987

[acel12424-bib-0502] Martin GM (2009) Epigenetic gambling and epigenetic drift as an antagonistic pleiotropic mechanism of aging. Aging Cell 8, 761–764. 10.1111/j.1474-9726.2009.00515.x 19732045

[acel12424-bib-0058] McGee MD , Weber D , Day N , Vitelli C , Crippen D , Herndon LA , Hall DH , Melov S (2011) Loss of intestinal nuclei and intestinal integrity in aging *C. elegans* . Aging Cell 10, 699–710. 10.1111/j.1474-9726.2011.00713.x PMC3135675 21501374

[acel12424-bib-0059] Melov S , Lithgow GJ , Fischer DR , Tedesco PM , Johnson TE (1995) Increased frequency of deletions in the mitochondrial genome with age of *Caenorhabditis elegans* . Nucleic Acids Res. 23, 1419–1425. 10.1093/nar/23.8.1419 PMC306871 7753635

[acel12424-bib-0060] Melov S , Schneider JA , Coskun PE , Bennett DA , Wallace DC (1999) Mitochondrial DNA rearrangements in aging human brain and in situ PCR of mtDNA. Neurobiol. Aging 20, 565–571. 10.1016/s0197-4580(99)00092-5 10638530

[acel12424-bib-0061] Mendenhall AR , Tedesco PM , Taylor LD , Lowe A , Cypser JR , Johnson TE (2012) Expression of a single‐copy hsp‐16.2 reporter predicts life span. J. Gerontol. A Biol. Sci. Med. Sci. 67, 726–733. 10.1093/gerona/glr225 PMC3391070 22227523

[acel12424-bib-0062] Mendenhall AR , Tedesco PM , Sands B , Johnson TE , Brent R (2015) Single cell quantification of reporter gene expression in live adult *Caenorhabditis elegans* reveals reproducible cell‐specific expression patterns and underlying biological variation. PLoS One 10, e0124289. 10.1371/journal.pone.0124289 PMC4422670 25946008

[acel12424-bib-0063] Miesenbock G , De Angelis DA , Rothman JE (1998) Visualizing secretion and synaptic transmission with pH‐sensitive green fluorescent proteins. Nature 394, 192–195. 10.1038/28190 9671304

[acel12424-bib-0064] Morley JF , Brignull HR , Weyers JJ , Morimoto RI (2002) The threshold for polyglutamine‐expansion protein aggregation and cellular toxicity is dynamic and influenced by aging in *Caenorhabditis elegans* . Proc. Natl Acad. Sci. USA 99, 10417–10422. 10.1073/pnas.152161099 PMC124929 12122205

[acel12424-bib-0065] Mortimer RK , Johnston JR (1959) Life span of individual yeast cells. Nature 183, 1751–1752. 10.1038/1831751a0 13666896

[acel12424-bib-0067] National Institute on Aging (2011) Biology of aging: research today for a healthier tomorrow, pp. 1 online resource (40 pages) in *NIH publication no. 11‐7561* Bethesda, MD: National Institute on Aging National Institutes of Health U.S. Department of Health and Human Services.

[acel12424-bib-0068] Nolte DD (2010) The tangled tale of phase space. Phys. Today 63, 33–38.

[acel12424-bib-0069] Ogg S , Paradis S , Gottlieb S , Patterson GI , Lee L , Tissenbaum HA , Ruvkun G (1997) The Fork head transcription factor DAF‐16 transduces insulin‐like metabolic and longevity signals in *C. elegans* . Nature 389, 994–999. 10.1038/40194 9353126

[acel12424-bib-0070] Pearl R (1928) The Rate of Living. New York: Alfred Knopf.

[acel12424-bib-0071] Rea SL , Wu D , Cypser JR , Vaupel JW , Johnson TE (2005) A stress‐sensitive reporter predicts longevity in isogenic populations of *Caenorhabditis elegans* . Nat. Genet. 37, 894–898. 10.1038/ng1608 PMC1479894 16041374

[acel12424-bib-0072] Rivlin R (1992) An individual's healthspan, to a great extent, is written in the genes In: Mirabela. New York: Hachette Filipacchi 4, pp. 152.

[acel12424-bib-0073] Rixen D , Siegel JH , Friedman HP (1996) “Sepsis/SIRS”, physiologic classification, severity stratification, relation to cytokine elaboration and outcome prediction in posttrauma critical illness. J. Trauma 41, 581–598. 10.1097/00005373-199610000-00001 8858015

[acel12424-bib-0074] Sands B , Brent R (2015) Overview of post‐Cohen‐Boyer methods for single segment cloning and for multisegment DNA assembly. Curr. Protoc. Mol. Biol., in press. 10.1002/0471142727.mb0326s113 PMC4853029 27152131

[acel12424-bib-0075] Sands B , Jenkins P , Peria WJ , Naivar M , Houston JP , Brent R (2014) Measuring and sorting cell populations expressing isospectral fluorescent proteins with different fluorescence lifetimes. PLoS One 9, e109940. 10.1371/journal.pone.0109940 PMC4193854 25302964

[acel12424-bib-0076] Seewald AK , Cypser J , Mendenhall A , Johnson T (2010) Quantifying phenotypic variation in isogenic *Caenorhabditis elegans* expressing Phsp‐16.2:gfp by clustering 2D expression patterns. PLoS One 5, e11426. 10.1371/journal.pone.0011426 PMC2906502 20657830

[acel12424-bib-0077] Shee C , Cox BD , Gu F , Luengas EM , Joshi MC , Chiu LY , Magnan D , Halliday JA , Frisch RL , Gibson JL , Nehring RB , Do HG , Hernandez M , Li L , Herman C , Hastings PJ , Bates D , Harris RS , Miller KM , Rosenberg SM (2013) Engineered proteins detect spontaneous DNA breakage in human and bacterial cells. Elife 2, e01222. 10.7554/eLife.01222 PMC3809393 24171103

[acel12424-bib-0078] Siegel JH , Goldwyn RM , Farrell EJ , Gallin P , Friedman HP (1974) Hyperdynamic states and the physiologic determinants of survival in patients with cirrhosis and portal hypertension. Arch. Surg 108, 282–292. 10.1001/archsurg.1974.01350270016004 4813331

[acel12424-bib-0079] Siegel JH , Cerra FB , Peters D , Moody E , Brown D , McMenamy RH , Border JR (1979) The physiologic recovery trajectory as the organizing principle for the quantification of hormonometabolic adaptation to surgical stress and severe sepsis. Adv. Shock Res. 2, 177–203. 318078

[acel12424-bib-0080] Silventoinen K , Magnusson PK , Tynelius P , Kaprio J , Rasmussen F (2008) Heritability of body size and muscle strength in young adulthood: a study of one million Swedish men. Genet. Epidemiol. 32, 341–349. 10.1002/gepi.20308 18271028

[acel12424-bib-0081] Spudich JL , Koshland DE Jr (1976) Non‐genetic individuality: chance in the single cell. Nature 262, 467–471. 10.1038/262467a0 958399

[acel12424-bib-0082] Strehler BL (1986) Genetic instability as the primary cause of human aging. Exp. Gerontol. 21, 283–319. 10.1016/0531-5565(86)90038-0 3545872

[acel12424-bib-0083] Stringari C , Cinquin A , Cinquin O , Digman MA , Donovan PJ , Gratton E (2011) Phasor approach to fluorescence lifetime microscopy distinguishes different metabolic states of germ cells in a live tissue. Proc. Natl Acad. Sci. USA 108, 13582–13587. 10.1073/pnas.1108161108 PMC3158156 21808026

[acel12424-bib-0084] Tantama M , Hung YP , Yellen G (2011) Imaging intracellular pH in live cells with a genetically encoded red fluorescent protein sensor. J. Am. Chem. Soc. 133, 10034–10037. 10.1021/ja202902d PMC3126897 21631110

[acel12424-bib-0085] Vijg J , Dolle ME (2002) Large genome rearrangements as a primary cause of aging. Mech. Ageing Dev. 123, 907–915. 10.1016/s0047-6374(02)00028-3 12044939

[acel12424-bib-0086] Vijg J , Suh Y (2013) Genome instability and aging. Annu. Rev. Physiol. 75, 645–668. 10.1146/annurev-physiol-030212-183715 23398157

[acel12424-bib-0087] Winter PW , Shroff H (2014) Faster fluorescence microscopy: advances in high speed biological imaging. Curr. Opin. Chem. Biol. 20, 46–53. 10.1016/j.cbpa.2014.04.008 PMC4096075 24815857

[acel12424-bib-0088] Zeug A , Woehler A , Neher E , Ponimaskin EG (2012) Quantitative intensity‐based FRET approaches – a comparative snapshot. Biophys. J . 103, 1821–1827. 10.1016/j.bpj.2012.09.031 PMC3491707 23199910

[acel12424-bib-0089] Zhao Y , Araki S , Wu J , Teramoto T , Chang YF , Nakano M , Abdelfattah AS , Fujiwara M , Ishihara T , Nagai T , Campbell RE (2011) An expanded palette of genetically encoded Ca(2)(+) indicators. Science 333, 1888–1891. 10.1126/science.1208592 PMC3560286 21903779

[acel12424-bib-0090] Zhou X , Clister TL , Lowry PR , Seldin MM , Wong GW , Zhang J (2015) Dynamic visualization of mTORC1 activity in living cells. Cell Rep. 10, 1767–1777. 10.1016/j.celrep.2015.02.031 PMC4567530 25772363

